# *Streptomyces coelicolor* SCO4226 Is a Nickel Binding Protein

**DOI:** 10.1371/journal.pone.0109660

**Published:** 2014-10-06

**Authors:** Mo Lu, Yong-Liang Jiang, Shu Wang, Hua Jin, Rong-Guang Zhang, Marie-Joelle Virolle, Yuxing Chen, Cong-Zhao Zhou

**Affiliations:** 1 Hefei National Laboratory for Physical Sciences at the Microscale and School of Life Sciences, University of Science and Technology of China, Hefei, Anhui, People's Republic of China; 2 Institute of Biophysics, Chinese Academy of Sciences, Beijing, China; 3 Institut de Génétique et Microbiologie, UMR8621 CNRS Université Paris Sud, Orsay, France; 4 Department of Biology, South University of Sciences and Technology of China, Shenzhen, Guangdong, People's Republic of China; Russian Academy of Sciences, Institute for Biological Instrumentation, Russian Federation

## Abstract

The open reading frame *SCO4226* of *Streptomyces coelicolor* A3(2) encodes an 82-residue hypothetical protein. Biochemical assays revealed that each SCO4226 dimer binds four nickel ions. To decipher the molecular function, we solved the crystal structures of SCO4226 in both apo- and nickel-bound (Ni-SCO4226) forms at 1.30 and 2.04 Å resolution, respectively. Each subunit of SCO4226 dimer adopts a canonical ferredoxin-like fold with five β-strands flanked by two α-helices. In the structure of Ni-SCO4226, four nickel ions are coordinated at the surface of the dimer. Further biochemical assays suggested that the binding of Ni^2+^ triggers the self-aggregation of SCO4226 *in vitro*. In addition, RT-qPCR assays demonstrated that the expression of *SCO4226* gene in *S. coelicolor* is specifically up-regulated by the addition of Ni^2+^, but not other divalent ions such as Cu^2+^, Mn^2+^ or Co^2+^. All these results suggested that SCO4226 acts as a nickel binding protein, probably required for nickel sequestration and/or detoxification.

## Introduction

Nickel is first demonstrated as a bacterial growth nutrient for *Hydrogenomonas* strains [1]. Since then studies on the physiological role of nickel have grown tremendously [2]. Nine types of enzyme, which utilize nickel as a catalytic cofactor have been identified, including hydrogenase, urease, superoxide dismutase (SOD), carbon monoxide dehydrogenase, acetyl-CoA decarbonylase/synthase, methyl-CoM reductase, glyoxylase I, acireductone dioxygenas and methylenediurease [3]. Nickel is also one of the most common contact sensitizers causing delayed-type hypersensitivity in humans [4]. Accordingly, the cellular immune response to nickel has become a model system for studying hapten-induced hypersensitivity [5]. However, the intracellular accumulation of nickel to millimolar level is toxic to cells, and inhibits the growth of most bacteria [6], which might be due to the induction of reactive oxygen species [7]. Therefore, microorganisms evolve a variety of transporters, sensor/regulators and storage proteins to maintain nickel homeostasis [8], [9].

Nickel is uptaken by the cells via two types of high-affinity importer systems, ABC transporters and the nickel/cobalt permeases [8], [10]. The nickel ABC transporter from *Escherichia coli* consists of five proteins (NikA-E), which transports nickel by coupling ATP hydrolysis [11]. The nickel permease (*HoxN*) from *Cupriavidus necator* was observed to transport nickel [12]. After nickel is transported into the cells, the nickel regulator, exporter and/or storage proteins are responsible to maintain the intracellular homeostasis of nickel. The best-characterized nickel sensor/regulator is NikR, which was first identified in *E. coli* [11]. The binding of nickel in the C-terminal metal-binding domain of NikR may trigger the conformational changes of its DNA binding domain to facilitate the interaction with a 28-bp palindromic DNA of the nickel uptake operon *NikABCDE* [13]. In addition, Nickel storage proteins could bind multiple nickel ions via the histidine-rich N-terminus [14]. For example, HypB in *Bradyrhizobium japonicum* can bind nine nickel ions per monomer at the N-terminal histidine-rich region [15], [16]. On the other hand, the exporters could pump superfluous nickel ions out of the cells [17]. For instance, the expression of a nickel exporter gene *nreB* was specifically induced by the presence of nickel and conferred nickel resistance in both *Achromobacter xylosoxidans* and *E. coli* [18].

*Streptomyces*, a genus of filamentous Gram-positive bacteria usually living in the superficial layers of the soil, are crucial for maintaining the microenvironment because of their broad range of metabolic processes and biotransformations [19], [20]. Several nickel-related proteins have been proposed to be involved in nickel homeostasis and regulation to avoid its cytotoxic effects [21]. The nickel-responsive transcription factor Nur in *Streptomyces coelicolor* has been proven to control the nickel homeostasis and anti-oxidative response through repressing the expression of a nickel-transporter gene cluster [22]. While the nickel-containing superoxide dismutases (NiSOD) have been identified in several *Streptomyces* species [23]. Moreover, addition of nickel ions can up-regulate the *NiSOD* gene and down-regulate the *FeSOD* gene in *S. coelicolor* [24]. However, the fine regulation of nickel homeostasis in *S. coelicolor* remains largely unclear. Here we showed that the 82-residue hypothetical protein SCO4226 is a nickel binding protein. Further structural and biochemical analyses revealed that SCO4226 could sequester the excess nickel ions via self-aggregation. These findings provided novel insights into the nickel response in *Streptomyces*.

## Materials and Methods

### Bacterial strains and culture conditions

*S. coelicolor* A3(2) strain was grown and maintained according to the standard procedures [25]. Gauze's medium No. 1 and YEME medium [YEME medium with yeast extract (Difco), 3 g/L; Bacto Peptone (Difco), 5 g/L; malt extract (Difco), 3 g/L; glucose, 10 g/L; sucrose, 340 g/L; and MgCl_2_.6H_2_0, 0.25 g/L] were used to cultivate the *S. coelicolor* A3(2) strain. Pre-germinated spores were inoculated into seed media and cultivated at 28°C for 24–48 hr. One percent (v/v) of the seed culture was inoculated to the main media and grown for 48 hr in YEME medium.

### Cloning, expression, purification, and Se-Met labeling

The coding region of *SCO4226* was cloned as described previously [26]. The recombinant plasmid was transformed into *E. coli* B834 (DE3). The cells were cultivated in seleno-Methionine (Se-Met)-M9 minimal medium (M9 medium with 1% glucose, 2 mM MgSO_4_, 0.1 mM CaCl_2_, 7.5 mg/L FeSO_4_, 1 µg/ml thiamine, 0.05 g/L for each amino acid except that methionine was replaced by 0.025 g/L Se-Met). The expression of the target protein was induced for 5 hr at 37°C by adding isopropyl-β-D-1-thiogalactopyranoside (IPTG) to a final concentration of 0.2 mM when OD_600nm_ reached 0.6. Following the purification procedures as reported previously [26], the target Se-Met substituted protein was purified by a two-step protocol: an affinity chromatography with nickel-chelating Sepharose fast flow column (Amersham Biosciences) followed by a gel filtration using a HiLoad 16/60 Superdex 75 column (Amersham Biosciences) equilibrated with the buffer containing 20 mM Tris-HCl, pH 7.5,100 mM NaCl. The target protein was pooled and the purity of the protein was verified by sodium dodecyl sulfate polyacrylamide gel electrophoresis (SDS-PAGE) and Liquid chromatography mass spectrometry (LTQ, Thermo Fisher).

### Crystallization, data collection and structure determination

The Se-Met labeled apo-SCO4226 (Se-apo-SCO4226) crystals were grown as described previously [26]. The Ni-SCO4226 crystals were obtained by soaking the Se-apo-SCO4226 crystals in the reservoir solution containing 0.8 M sodium citrate dihydrate, pH 6.5 and 0.8 M NiSO_4_ for about 1 hr. All crystals were flash cooled in liquid nitrogen using reservoir solution containing 25% (v/v) glycerol as the cryoprotectant. The diffraction data of Se-apo-SCO4226 were collected at the synchrotron radiation source at the Structural Biology Center, Argonne National Laboratory (Argonne, USA) and were processed with HKL2000 [27] and SCALEPACK [28]. The Ni-SCO4226 data were recorded at 100 K in a liquid nitrogen stream using a Rigaku MM007 X-ray generator (λ = 1.5418 Å) with a MarRearch 345 image-plate detector at School of Life Sciences, University of Science and Technology of China (USTC, Hefei, China) and were processed with the program MOSFLM 7.0.4 [29]. The apo-SCO4226 structure was determined by single wavelength anomalous dispersion (SAD) method [30]. The Ni-SCO4226 structure was determined by the molecular replacement method using the structure of apo-SCO4226 as a search model with the program MOLREP [31] in CCP4i [32]. The initial models were refined by using the maximum likelihood method with the program REFMAC5 [33] and rebuilt interactively with the programs PHENIX [34] and COOT [35] until the free R-factor converged. The occupancy refinement with PHENIX was used to refine the individual occupancies of nickel ions and group occupancies of citrate in Ni-SCO4226. The final models were checked and validated using the programs MOLPROBITY [36] and PROCHECK [37]. Data collection and refinement statistics were listed in Table 1.

**Table 1:** Crystal parameters, data collection and structure refinement.

	apo-SCO4226	Ni-SCO4226
*Data collection*		
Space group	*P2_1_*	*P2_1_*
Unit cell (Å)	29.57, 66.98, 34.27	28.33, 67.14, 35.09
Unit cell (°)	90.00, 95.02, 90.00	90.00, 94.98, 90.00
Resolution range (Å)	34.14–1.30 (1.32–1.30)^a^	28.23–2.03 (2.14–2.04)
Unique reflections	30,687 (1,081)	8,364 (1,088)
Completeness (%)	93.9 (66.6)	98.3 (88.6)
*<I/σ(I)>*	19.70 (3.34)	19.7 (8.1)
R_merge_ b (%)	10.4 (31.7)	4.8 (16.1)
Average redundancy	5.0 (4.1)	3.6 (3.5)
*Structure refinement*		
Resolution range (Å)	34.14–1.30	28.23–2.04
R-factor^c^/R-free^d^ (%)	15.2/16.9	16.9/21.8
Number of protein atoms	1276	1263
Number of water atoms	158	86
RMSD^e^ bond lengths (Å)	0.007	0.010
RMSD bond angles (°)	1.073	1.164
Mean B factors (Å^2^)	9.95	15.78
Ramachandran plot^f^ (residues,%)		
Most favored (%)	97.47	98.10
Additional allowed (%)	2.53	1.90
Outliers (%)	0	0
PDB entry	4OI3	4OI6

a The values in parentheses refer to statistics in the highest bin.

b R_merge_ = ∑_hkl_∑_i_|I_i_(hkl)−<I(hkl)>|/∑_hkl_∑_i_I_i_(hkl), where I_i_(hkl) is the intensity of an observation and <I(hkl)> is the mean value for its unique reflection; Summations are over all reflections.

c R-factor  =  ∑_h_||Fo(h)|−|Fc(h)||/∑_h_|Fo(h)|, where |Fo| and |Fc| are the observed and calculated structure-factor amplitudes, respectively.

d R-free was calculated with 5% of the data excluded from the refinement.

e Root-mean square-deviation from ideal values.

f Categories were defined by Molprobity.

### Isothermal titration calorimetry

The chemical NiSO_4_ was bought from Sangon, China. The titrations of Ni^2+^ into the SCO4226 protein solution were performed at 28°C using VP-ITC Isothermal Titration Calorimeter (ITC, MicroCal Inc.). Before each ITC experiment, the pH values were adjusted to the same, and the protein solution was degassed for 2–5 min to eliminate air bubbles. In each individual titration, the 40 µL NiSO_4_ solution of varying concentrations (200–500 µM) was injected into the SCO4226 protein solution (10–20 µM) in the same buffer containing 20 mM Tris-HCl, pH 7.0, 100 mM NaCl. A 180 sec interval between injections was used for complete equilibrium. Titration of Ni^2+^ into the buffer alone under the same conditions was used as the control. The data were fitted using a non-linear least squares minimization algorithm to a theoretical titration curve, using the MicroCal Origin software. Δ*H* (reaction enthalpy change in cal mol^−1^), *K*_b_ (binding constant in M^−1^), and *n* (number of binding sites) were the fitting parameters. The reaction entropy was calculated using the relationships Δ*G*  =  −*RT*ln *K*_b_ (R = 1.9872 cal mol^−1^ K^−1^, T = 298 K) and Δ*G*  =  Δ*H* − TΔ*S*. The reduced chi-square parameter χ_v_^2^ (χ_v_^2^  =  χ^2^/*n*, where *n* is the degrees of freedom, *n*  =  *N*_idp_ − *N*_par_, *N*_idp_  =  number of points, and *N*_par_  =  number of parameters floating in the fit) was used to establish the best fit. Best fits were obtained using the one set of site scheme for the Ni^2+^ binding curve.

### Dynamic Light Scattering Assays

Dynamic Light Scattering (DLS) assays were carried out on a DYNAPRO-99 (Wyatt Technology Corp., 6300 Hollister). The molecular size of the protein was determined by DLS using cylindrical light scattering cuvettes at 28°C. Determinations were conducted on an ALV/DLS/SLS-5022F spectrometer equipped with a multi-digital time correlation (ALV5000) and a cylindrical 22 mW He-Ne laser (λ_0_ = 632 nm, Uniphase) as the light source. The intensity-intensity time correlation function G^(2)^(t, q) was measured to determine the line-width distribution G(Γ) in dynamic LLS. For diffusive relaxation, Γ is related to the translational diffusion coefficient (*D*) of the scattering object in dilute solution or dispersion by *D*  =  (〈Γ〉/q^2^)_C→0,q→0_ and further to hydrodynamic radius (*R*_h_) from Stokes-Einstein equation: *R*_h_  =  k_B_T/(6πηD), where η, k_B_ and T are the solvent viscosity, the Boltzmann constant and the absolute temperature, respectively. Hydrodynamic radius distribution *f*(*R*_h_) was calculated from the Laplace inversion of G^(2)^(t, q) using the CONTIN program.

### UV/visible spectroscopy

The chemical NiSO_4_ and Ethylene Diamine Tetraacetic Acid (EDTA) were bought from Sangon, China. The turbidity of the protein solution towards NiSO_4_ was measured at 28°C by monitoring the change in absorbance at 340 nm in 30 min or more with the interval of 15 sec, using a DU800 spectrophotometer (Beckman Coulter, Fullerton, CA, USA). Titrations of NiSO_4_ (0–2 mM) against 10–50 µM SCO4226 were performed in the buffer of 0.1 M Tris-HCl, pH 7.5, 100 mM NaCl. Then EDTA was added to a final concentration of 5 mM in the turbidity solution. The data were recorded by the program Origin 7.5. The assays were performed in triplicates.

### RNA isolation

The bacteria of *S. coelicolor* A3(2) strain were grown in the YEME media with the addition of varying concentrations (100, 200 and 300 µM) of NiCl_2_, MnCl_2_, CuCl_2_ or CoCl_2_. The strain with the addition of buffer alone was used as the control. All chemicals of >98% purity were purchased from Sangon, China. RNA was isolated from the mycelia of *S. coelicolor* A3(2) strains which was grown for 48 hr on YEME medium by using an Spin Column Bacterial total RNA Purification Kit and the suggested protocol of the manufacturer (Sangon). The genomic DNA was removed by incubating one unit of RNAse-Free DNase I (Thermo) per 1 µg estimated DNA for 10 min at 37°C. The concentrations of RNA in different samples were calculated by determining the absorbance at 260 and 320 nm using a DU800 spectrophotometer (Beckman Coulter, Fullerton, CA, USA). The quality of RNA sample was assessed by electrophoresis in a formaldehyde denaturing agarose gel stained with ethidium bromide. Preliminary time course studies (data not shown) indicated that transcript levels were highest in 48 hr RNA preparations.

### Quantitative Real-time PCR (RT-qPCR) analysis

The cDNAs for RT-qPCR analyses were synthesized using PrimeScript RT reagent Kit (Perfect Real Time, TaKaRa Dalian Biotechnology Co., Ltd. Dalian, China). Quantitative Real-time PCR (RT-qPCR) assays were performed by the SYBR Premix Ex Taq II Kit (TliRnaseH Plus, TaKaRa Dalian Biotechnology Co., Ltd. Dalian, China) on ABI Stepone Real-Time PCR system in MicroAmp fast optical 48-well plates (Applied Biosystems). The expression of the *Streptomyces hrdB* gene, thought to encode a constitutively expressed vegetative sigma factor, was monitored as a control. A forward primer (5′-AAG GCG ACG GCG AAG AAG-3′) and a reverse primer (5′-TCG GCG TTG AGC AGA GGG AC-3′) were used for *hrdB* (272 bp). The primers used for RT-PCR analysis were designed as a forward primer (5′-CAC GGC ATC ACC TCG GAC CA-3′) and a reverse primer (5′-CGA CAG GGG GAC TTC GTG GA-3′) for *SCO4226* (210 bp). Each RT-qPCR experiment was carried out from at least three independent RNA biological samples and at least three independent experiments were done from each RNA sample.

## Results and Discussion

### Overall Structure

**Figure 1:**
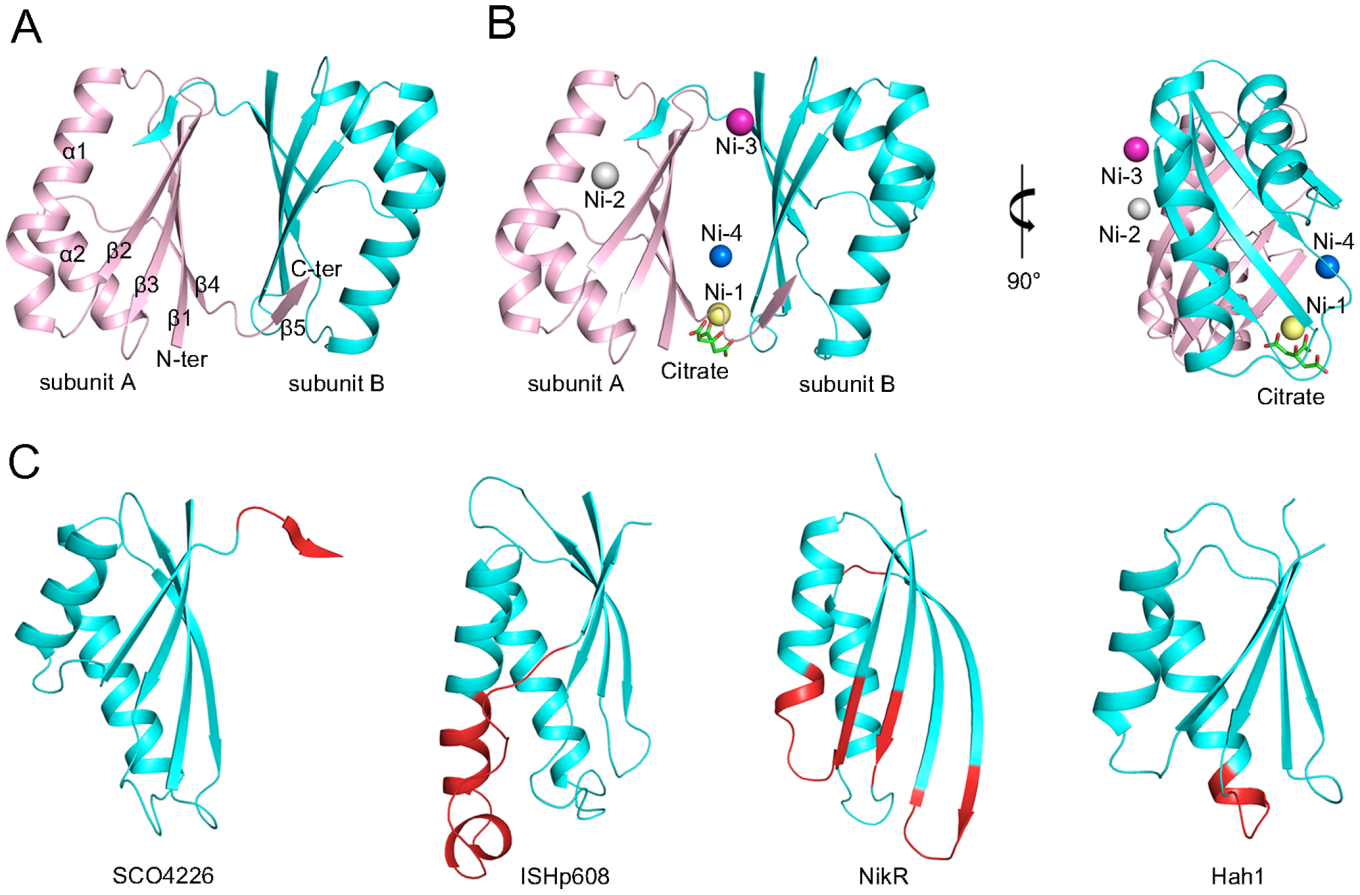
Overall structures of SCO4226 in (A) apo- and (B) nickel-bound forms (pink and cyan for each subunit, respectively). The Ni-SCO4226 structure is presented in two perpendicular orientations. The citrate molecule is shown as green sticks and the Ni^2+^ ions are shown as spheres (yellow for Ni-1, gray for Ni-2, purple for Ni-3 and blue for Ni-4). **(C)** Structural comparisons between one subunit of SCO4226, *Helicobacter pylori* transposase IS*Hp608* (PDB: 2A6M), *H. pylori* nickel-dependent transcription factor NikR (PDB: 3QSI), and *Homo sapiens* copper chaperone Hah1 (PDB: 1FE4). The four structures are shown in the same superimposed orientation. Residues of IS*Hp608*, NikR and Hah1 that can be superimposed with SCO4226 are colored cyan whereas others are colored red.

The structures of SCO4226 in both apo- and nickel-bound forms (termed apo- and Ni-SCO4226, respectively) have been solved at 1.30 and 2.04 Å resolution, respectively (PDB IDs: 4OI3 and 4OI6). The two structures are quite similar to each other with a root-mean-square deviation (RMSD) of 0.48 Å over all the 162 C*α* atoms. Each asymmetric unit contains a dimer of SCO4226, with a buried interface area of about 1100 Å^2^ on each subunit. The two subunits of the dimer are almost identical, with an RMSD of 0.14 Å for apo-SCO4226, and 0.29 Å for Ni-SCO4226. Size-exclusion chromatography in our previous study also indicated that SCO4226 exists as a dimer in solution [26]. The overall structure of SCO4226 adopts a ferredoxin-like fold [38] with an α+β structure: five β-strands (β1-5) packing against two α-helices (α1-2) (Figure 1A-1B). The N-terminal four β-strands β1-4 of one subunit form an antiparallel β-sheet, whereas the C-terminal β5 swaps to the corresponding β-sheet (β1′-4′) of the symmetric subunit, completing an extended antiparallel β-sheet of five strands. These two extended β-sheets face against each other to form a dimer interface, which possesses a hydrophobic core sealed by two hydrophilic laterals. The hydrophobic core is formed by residues Met5, Trp41, Tyr50, Leu52, and Val78 from both subunits. One of the laterals is mainly composed of hydrogen bonds between Arg9-Nη1 and Glu74′-Oε2, Arg9-Nη2 and Glu74′-Oε1, Asp43-Oδ1 and Glu77′-Oα, Tyr50-Oη and His76′-Nδ1, Glu74-Oε2 and Arg9′-Nη1, Glu74-Oε1 and Arg9′-Nη2, His76-Nδ1 and Tyr50′-Oη, Glu77-Oα and Asp43′-Oδ1, whereas the other one consists of backbone hydrogen bonds (Ala40-Oα and Ala82′-Nα, Ala42-Oα and Leu80′-Nα, Ala42-Nα and Leu80′-Oα) from strands β2 and β5′, and vice versa (residues from the symmetric subunit were labeled with a prime). In the structure of Ni-SCO4226, four nickel ions are assigned at the dimer interface, with Ni-1 and Ni-4 on one side, and Ni-2 and Ni-3 on the other (Figure 1B). In addition, one citrate molecule is bound to Ni-1 via coordinate bonds.

### Structural comparison

Structural similarity search using DALI server (http://ekhidna.biocenter.helsinki.fi/dali_server/) [39] gave 266 hits of 58 unique proteins with a Z-score of ≥6.0. The top hits include DNA transposase IS*Hp*608 from *Helicobacter pylori* (PDB: 2A6M, Z-score 7.7, RMSD 2.6 Å for 76 Cα atoms), the nickel-dependent transcription factor NikR from *H. pylori* (PDB: 3QSI, Z-score 7.3, RMSD 2.9 Å for 71 Cα atoms), and the metallochaperone protein Hah1 from *Homo sapiens* (PDB: 1FE4, Z-score 6.0, RMSD 2.4 Å for 63 Cα atoms).

Superposition of subunit A of the apo-SCO4226 structure against the ferredoxin-like fold domain of these structures revealed that the overall structures could be superimposed, except for the extension of β5 at the C-terminus of SCO4226 (Figure 1C). Nevertheless, significant structural variations were found between SCO4226 and these proteins. Specifically, the transposase IS*Hp*608 is dimeric, and binds two DNA stem loops representing the conserved inverted repeats near each the end of IS*Hp608* [40]. As compared to the DNA recognition motif of IS*Hp*608, the helix α2 of SCO4226 is 7-residue shorter than the counterpart helix αB of IS*Hp*608, which contributes to DNA binding. The nickel-dependent transcription factor NikR is a tetrameric protein that consists of a central tetrameric metal-binding domain (MBD) and two flanking ribbon-helix-helix DNA-binding domains (DBD) [41]. The MBD of NikR contains four nickel-binding sites at the tetramer interface [42], [43]. Comparing to the MBD of NikR, the β-sheet (β1-4) and two α-helices (α1-2) of SCO4226 show variations in length. In addition, SCO4226 is a dimer whereas the MBD of NikR is a tetramer. Notably, the Hah1 metallochaperone protein is implicated in copper delivery to the Menkes/Wilson disease proteins. Similar to SCO4226, Hah1 also adopts a ferredoxin-like fold, and belongs to a family of metal binding domains with a conserved MT/HCXXC motif [44]. However, SCO4226 lacks a conserved MT/HCXXC motif, indicating that it should adopt a different metal-binding pattern.

### Nickel coordination pattern

**Figure 2:**
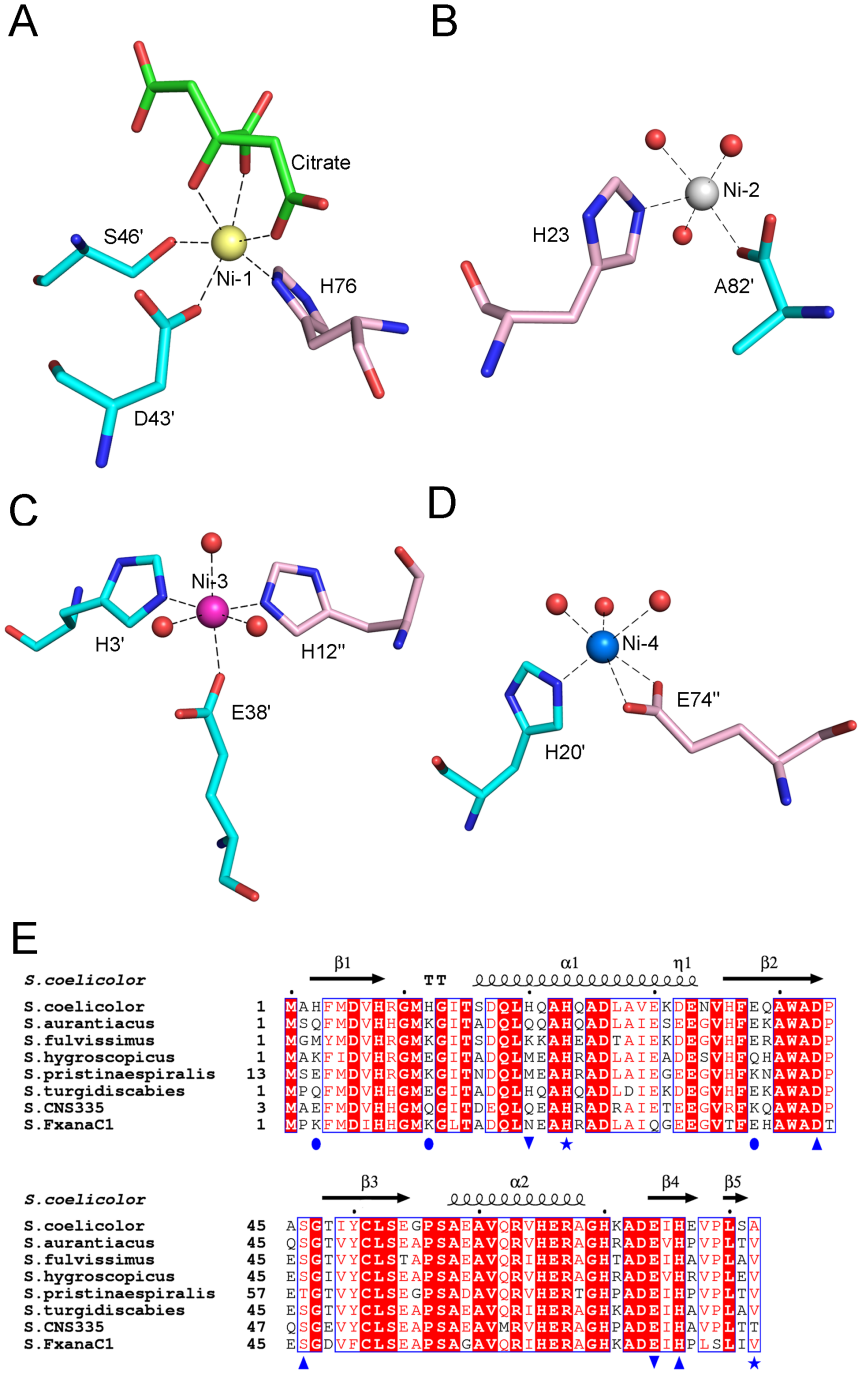
The coordination patterns of (A) Ni-1, (B) Ni-2, (C) Ni-3 and (D) Ni-4. The Ni^2+^ ions are shown as spheres and colored the same as **Figure 1B**. Black dotted lines donate the coordinate bonds. The coordinate residues and the citrate ion are shown in sticks. **(E)** Multiple sequence alignment of SCO4226 and homologs in *Streptomyces*. The secondary structural elements of *Streptomyces coelicolor* SCO4226 were labeled on the top. The nickel coordinate residues were labeled with different blue marks [Ni-1 (▴), Ni-2 (★), Ni-3 (•), Ni-4 (▾)]. Colors are chosen according to rules of ESPript (http://espript.ibcp.fr/ESPript/ESPript/): A blue frame represents a similarity across groups; a red character indicates similarity in a group; and a red box, white character demonstrates strict identity. All sequences were downloaded from the NCBI database (www.ncbi.nlm.nih.gov). The sequences are (NCBI accession numbers codes in parentheses) *Streptomyces aurantiacus* hypothetical protein (WP_016642010), *Streptomyces fulvissimus DSM 40593* DUF4242 domain containing protein (YP_007933639), *Streptomyces hygroscopicus subsp. jinggangensis 5008* hypothetical protein SHJG_4867 (YP_006246008), *Streptomyces pristinaespiralis* gualylate cyclase (WP_005311437), *Streptomyces turgidiscabies* hypothetical protein (WP_006377553), *Streptomyces sp. CNS335* gualylate cyclase (WP_018842673), *Streptomyces sp. FxanaC1* gualylate cyclase (WP_018089771).

In the dimeric structure of Ni-SCO4226, four nickel ions and one citrate molecule were identified in the electron density map. These four nickel ions are all surface exposed on the dimer interface with varying occupancies from 0.82 to 0.98. Our ITC data confirmed that four nickel ions are bound in one SCO4226 dimer with the *K_d_* value of 10 µM (Figure S1). All the four nickel ions are placed in the dimer interface. Ni-1 and Ni-2 interact with residues within one dimer whereas Ni-3 and Ni-4 are coordinated by residues from two adjacent dimers by symmetric operation. Specifically, Ni-1 is hexa-coordinated by His76-Nδ1, Asp43′-Oδ2, Ser46′-Oγ, and the O4, O7 and O6 atoms of the citrate molecule (Figure 2A). Ni-2 is penta-coordinated by His23-Nε2, Ala82′-OXT, and three water molecules (Figure 2B). Ni-3 of an octahedral geometry is hexa-coordinated by His3′-Nε2 and Glu38′-Oε2 of one dimer, His12″-Nε2 of the adjacent dimer, and three water molecules (residues from subunit A of the adjacent dimer were labeled with a double prime) (Figure 2C). Ni-4 coordination is formed by His20′-Nε2 of one dimer, Glu74″-Oε1, Glu74″-Oε2 of the adjacent dimer and three water molecules (Figure 2D). The distances for all coordinate bonds are well within the expected range (2.06-2.46 Å). Sequence analysis revealed that only nickel-binding residues His23, Asp43, Ser46, Glu74 and His76 are exclusively conserved in *Streptomyces*, among which, Asp43, Ser46, and His76 constitute the complete Ni-1 coordination (Figure 2E). This suggests that the Ni-1 binding site might be conserved whereas others should be plastic.

### *In vitro* aggregation of SCO4226 upon addition of excess nickel

**Figure 3:**
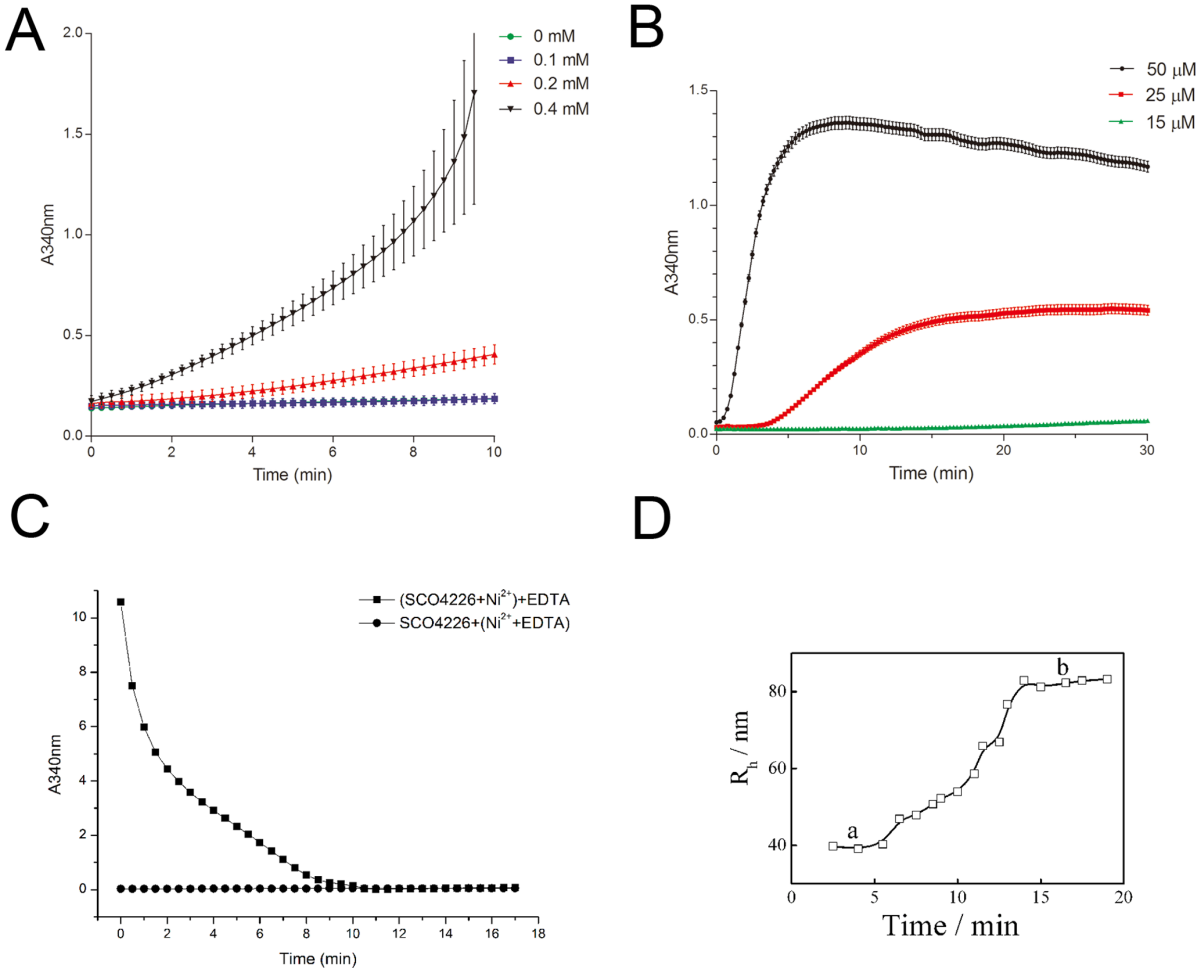
The aggregation of SCO4226 triggered by nickel. **(A)** UV/visible absorbance spectra (A_340nm_) of 50 µM SCO4226 protein solution with the addition of 0, 0.1, 0.2, 0.4 mM Ni^2+^ respectively in the buffer of 0.1 M Tris-HCl, pH 7.5, 100 mM NaCl at 28°C. **(B)** The A_340nm_ spectra of SCO4226 (15, 25 and 50 µM) in the buffer of 0.1 M Tris-HCl, pH 7.5, 100 mM NaCl upon the addition of 1 mM NiSO_4_ at 28°C. **(C)** The A_340nm_ spectra of Ni-SCO4226 protein solution with the addition of 5 mM EDTA. The A_340nm_ spectra of SCO4226 protein solution with 1 mM Ni^2+^ that has been pre-treated with 5 mM EDTA. **(D)** DLS detection of the particle radius of 50 µM SCO4226 protein solution with the addition of 1 mM Ni^2+^. The lower and upper plateaus are labeled with **a** and **b**, respectively.

As the absorbance at 340 nm (A_340nm_) could be used to indicate the turbidity of protein solution, the high level of which usually reflects the protein aggregation [45]. Thus we applied the change of A_340nm_ to explore the behavior of SCO4226 in solution upon binding to Ni^2+^. Without the addition of Ni^2+^, the A_340nm_ values are approximately zero, which indicated that the SCO4226 protein solution would not aggregate in the absence of Ni^2+^ (Figure 3A). However, the A_340nm_ values gradually increased upon the addition of Ni^2+^ at varying concentrations (Figure 3A). Moreover, the A_340nm_ values increased and gradually reached a plateau, resulted from the addition of 1 mM Ni^2+^ to the SCO4226 protein solution of varying concentrations (Figure 3B). Accompanied with the increase of the concentration of either SCO4226 or Ni^2+^, the A_340nm_ values sharply increased, which indicated the formation of more aggregates (Figure 3A-3B). These results suggested that the excess Ni^2+^ would trigger the aggregation of SCO4226. Notably, the addition of 5 mM EDTA to the SCO4226 solution in the presence of nickel led to an exponentially decrease of the A_340nm_ value (Figure 3C), demonstrating that Ni^2+^ binding is reversible. However, nickel pre-treated with EDTA will not trigger the aggregation of SCO4226 any more (Figure 3C). It suggested that SCO4226 could be restored once the excess Ni^2+^ ions are depleted.

Moreover, we used DLS experiments to detect the size of SCO4226 protein in solution at a concentration of 50 µM. The initial radius of SCO4226 molecule is approximately 1.2 nm. Upon the addition of 1 mM Ni^2+^, the SCO4226 molecules aggregated immediately and reached a lower plateau with an average particle radius of 40.0 nm (Figure 3D, plateau a). After incubation for 15 min, SCO4226 molecules kept on accumulating to form larger particles and finally stopped at an upper plateau with a maximum radius of 80.0 nm (Figure 3D, plateau b). These results further demonstrated that the nickel-triggered aggregation of SCO4226 is a dynamic process.

### The expression of *SCO4226* gene is up-regulated by nickel

**Figure 4:**
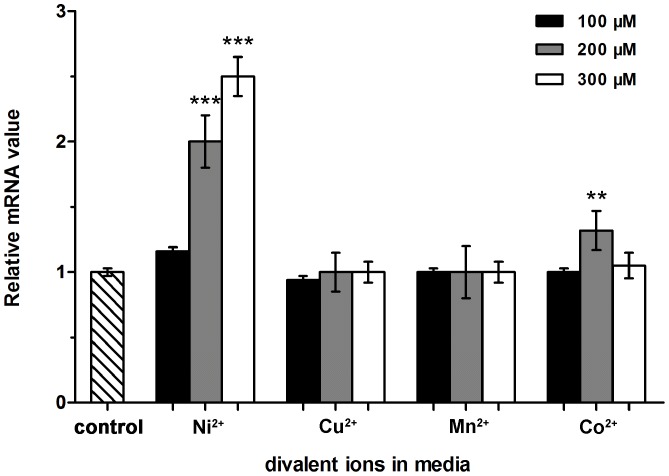
RT-qPCR analysis of the expression of *SCO4226* gene in *Streptomyces coelicolor* A3(2) strain. The strains were grown for 48 hr on YEME medium with different divalent ions (Ni^2+^, Cu^2+^, Mn^2+^ and Co^2+^) at three concentrations (100, 200 and 300 µM). The expression level of *SCO4226* gene without the addition of any metal ions was used as a control. Data are presented as the means ± standard deviations from three independent assays. One-way ANOVA with a post hoc Dunnett test is used for the comparison of statistical significance. The P values of <0.05, 0.01 and 0.001 are indicated with *, ** and ***, respectively.

To check if the *in vivo* expression of SCO4226 in *S. coelicolor* A3(2) is responsive to nickel, we compared the expression levels of *SCO4226* gene with the addition of Ni^2+^ or other divalent ions (Mn^2+^, Cu^2+^ or Co^2+^) at the concentration of 100, 200 and 300 µM, respectively. The total RNA was isolated from *S. coelicolor* A3(2) strain after 48 hr incubation. The expression levels of *SCO4226* gene were assessed by RT-qPCR assays. The addition of 100 µM Ni^2+^ increased the expression levels of *SCO4226* gene to about 1.2 folds, as compared to the control without the addition of any metal ions. Accompanied with the increase of Ni^2+^ concentration to 200 and 300 µM, the expression levels of *SCO4226* gene were gradually increased to 2 and 3 folds, respectively (Figure 4). In contrast, the addition of Mn^2+^, Cu^2+^, and Co^2+^ did not change the expression levels of *SCO4226*. The results indicated that the expression of *SCO4226* gene is specifically up-regulated by the addition of Ni^2+^, but not other divalent ions such as Cu^2+^, Mn^2+^ or Co^2+^.

### SCO4226 represents a novel nickel binding protein

The overall structure of SCO4226 reveals a ferredoxin-like fold with four nickel ions coordinated at the dimer interface. Each nickel ion has oxygen-containing ligands like water molecules and at least one histidine ligand (Figure 2), which is consistent with the notion that histidine is a common nickel ligand [46]. Although the crystal was soaked in a buffer of NiSO_4_ as high as 0.8 M, the four nickel-binding sites have an occupancy from 0.82 to 0.98. In fact, SCO4226 possesses a rather low affinity towards nickel, for all nickel-binding sites are solvent-exposed and utilize a few coordinate residues. However, despite lacking a conserved metal-binding motif, the abundance of metal-binding residues (eight glutamates, six aspartates and nine histidines) enables SCO4226 to bind multiple nickel ions. Similar cases have been also found in *E. coli* NikRs. The excess nickel binds to a total of 22 possible low-affinity nickel sites on the NikR tetramer. These sites are all on the surface of NikR, and most of them can be described as preferring octahedral geometry, utilizing one to three protein ligands (typically histidine) and at least two water molecules [47]. Despite the similarity of the multiple nickel-binding sites between *E. coli* NikR and SCO4226, further investigation are still needed for elucidating the relevance of nickel binding to the physiological function of SCO4226.

Our data showed that SCO4226 reversibly binds multiple nickel ions with low affinity. Similar cases have also been found in several proteins with multiple metal-binding sites. For instance, *H. pylori* histidine-rich protein Hpn plays a pivotal role in Ni^2+^ homoeostasis, binding or transport [48]. *B. japonicum* HypB with a histidine-rich region can bind multiple nickel ions and is thought to be involved in nickel storage [14], [15]. In addition, *A. xylosoxidans* NreB with a histidine-rich C-terminus is specifically induced by nickel and confers nickel resistance and reduces nickel accumulation upon heterologous expression in *E. coli* [18]. Notably, the metal-binding capabilities of these proteins are comparable to that of SCO4226 towards Ni^2+^. We thus propose that SCO4226 might also be involved in cellular nickel homeostasis, detoxification, and/or nickel utilization in specialized cytoplasmic compartments. On the other hand, the increase of cytoplasmic Ni^2+^ concentration triggers the aggregation of SCO4226, and in turn decreases its intracellular concentration. Thus, the up-expression of *SCO4226* gene might be a physiological response to guarantee the constant level of functioning SCO4226. Altogether, we proposed that SCO4226 is capable of sequestering excess nickel for fine-tuning the nickel homeostasis. However, the bona fide molecular and cellular function of SCO4226 remains to be investigated.

## Supporting Information

Figure S1 **ITC titration data for the binding of apo-SCO4226 with nickel.** The assays were performed at 28°C in the buffer of 20 mM Tris-HCl, pH 7.5. Raw titration data represent the thermal effect of 40 µL injections of Ni^2+^ (500 µM) onto the protein solution (20 µM). The continuous lines represent the best fit of the integrated data, obtained by a non-linear least squares procedure. The calculated number of binding sites and dissociation constant are indicated.
(TIF)
