# Supplementary figures and images for: Streptomyces coelicolor SCO4226 Is a Nickel Binding Protein
Source: PLoS One. 2014 Oct 6;9(10):e109660. doi: 10.1371/journal.pone.0109660 (PMC4186839; doi:10.1371/journal.pone.0109660)

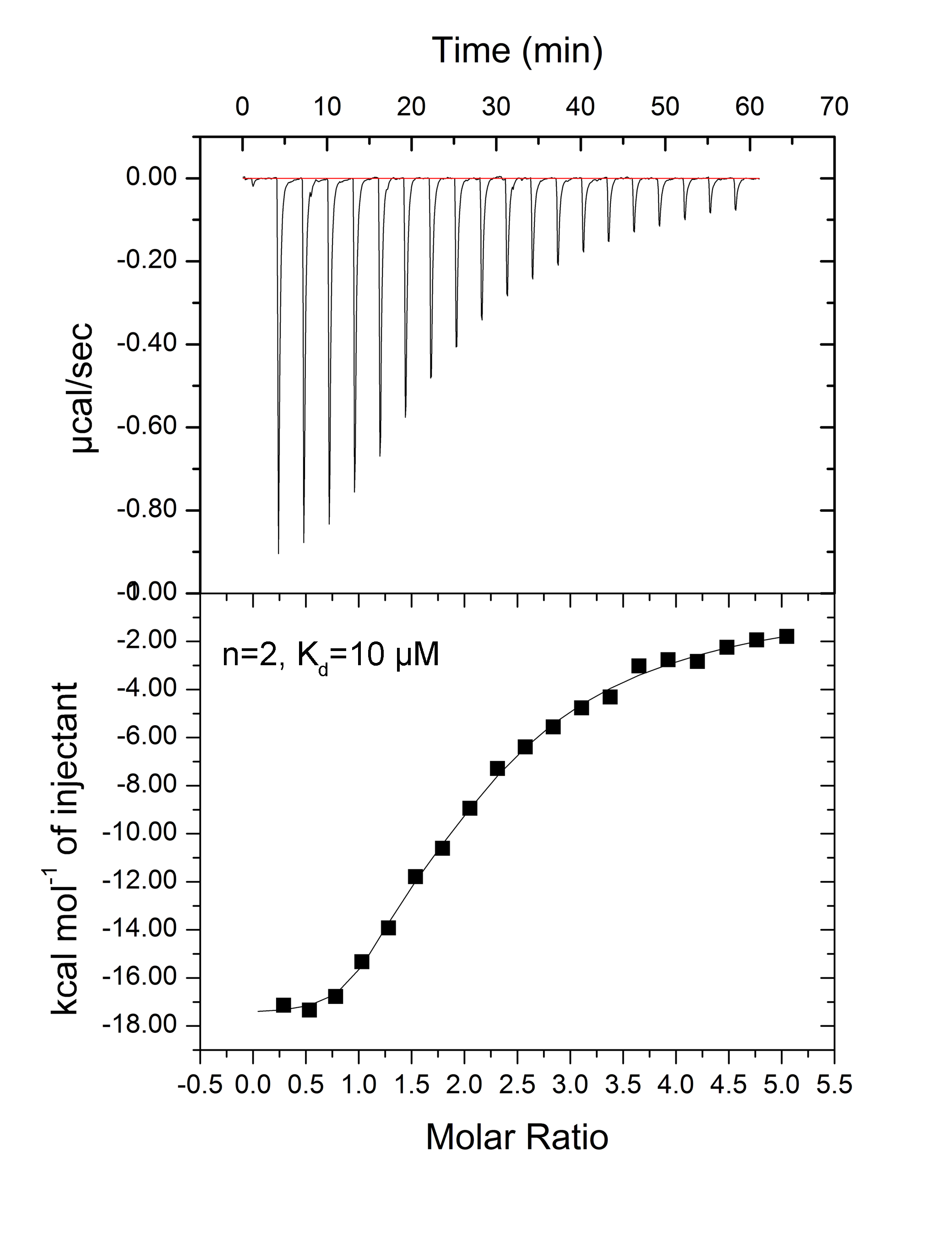

Supplement: Figure S1 — ITC titration data for the binding of apo-SCO4226 with nickel. The assays were performed at 28°C in the buffer of 20 mM Tris-HCl, pH 7.5. Raw titration data represent the thermal effect of 40 µL injections of Ni2+ (500 µM) onto the protein solution (20 µM). The continuous lines represent the best fit of the integrated data, obtained by a non-linear least squares procedure. The calculated number of binding sites and dissociation constant are indicated. (TIF) [file pone.0109660.s001.tif]
